# Melanesia holds the world’s most diverse and intact insular amphibian fauna

**DOI:** 10.1038/s42003-022-04105-1

**Published:** 2022-11-04

**Authors:** Paul M. Oliver, Deborah S. Bower, Peter J. McDonald, Fred Kraus, Jennifer Luedtke, Kelsey Neam, Louise Hobin, Alienor L. M. Chauvenet, Allen Allison, Evy Arida, Simon Clulow, Rainer Günther, Elizah Nagombi, Burhan Tjaturadi, Scott L. Travers, Stephen J. Richards

**Affiliations:** 1 Centre for Planetary Health and Food Security, Griffith University, Brisbane, Queensland 4121 Australia; 2 Biodiversity and Geosciences Program, Queensland Museum, South Brisbane, Queensland 4101 Australia; 3 Zoology Discipline, School of Environmental and Rural Science, University of New England, Armidale, NSW 2351 Australia; 4 Flora and Fauna Division, Department of Environment, Parks and Water Security, Alice Springs, NT 0870 Australia; 5 Department of Ecology and Evolutionary Biology, University of Michigan, Ann Arbor, MI 48109 USA; 6 IUCN SSC Amphibian Specialist Group, 3701 Lake Shore Blvd W, P.O. Box 48586, Toronto, Ontario M8W 1P5 Canada; 7 Re:wild, P.O. Box 129, Austin, Texas 78767 USA; 8 Bishop Museum, 1525 Bernice Street, Honolulu, HI 96817 USA; 9 Division of Zoology, Research Center for Biology, Indonesian Institute of Sciences (LIPI), Cibinong, Indonesia; 10 Centre for Conservation Ecology and Genomics, Institute for Applied Ecology, University of Canberra, Bruce, ACT 2617 Australia; 11 Museum für Naturkunde, Berlin, D-10115 Germany; 12 Wildlife Conservation Society, Goroka, Eastern Highlands Province Papua New Guinea; 13 Center for Environmental Studies, Sanata Dharma University (CESSDU), Yogyakarta, Indonesia; 14 Department of Biological Sciences, Rutgers University-Newark, Newark, NJ 07102 USA; 15 Herpetology Department, South Australian Museum, Adelaide, S.A. 5000 Australia

**Keywords:** Conservation biology, Biogeography, Tropical ecology, Herpetology

## Abstract

Identifying hotspots of biological diversity is a key step in conservation prioritisation. Melanesia—centred on the vast island of New Guinea—is increasingly recognised for its exceptionally species-rich and endemic biota. Here we show that Melanesia has the world’s most diverse insular amphibian fauna, with over 7% of recognised global frog species in less than 0.7% of the world’s land area, and over 97% of species endemic. We further estimate that nearly 200 additional candidate species have been discovered but remain unnamed, pointing to a total fauna in excess of 700 species. Nearly 60% of the Melanesian frog fauna is in a lineage of direct-developing microhylids characterised by smaller distributions than co-occurring frog families, suggesting lineage-specific high beta diversity is a key driver of Melanesian anuran megadiversity. A comprehensive conservation status assessment further highlights geographic concentrations of recently described range-restricted threatened taxa that warrant urgent conservation actions. Nonetheless, by world standards, the Melanesian frog fauna is relatively intact, with 6% of assessed species listed as threatened and no documented extinctions; and thus it provides an unparalleled opportunity to understand and conserve a megadiverse and relatively intact insular biota.

## Abstract (web summary)

Melanesia, centred on the vast tropical island of New Guinea, is shown to have the most diverse and intact insular frog fauna in the world.

## Introduction

Identifying concentrations of species richness, endemism and extinction threats are important steps in prioritising limited scientific resources to aid biodiversity conservation^1–4^. However, the availability of biological data varies regionally, and many of the most biodiverse jurisdictions are the most lacking in scientific research^5^. The Melanesian region encompasses hundreds of islands extending from New Guinea (and adjacent land-bridge islands along its western margin) east to Fiji and is an area of exceptional biocultural diversity^6^. New Guinea is the world’s largest tropical island, and its size combined with a complex topography and geology have generated the richest island flora in the world^7^. To the north and east of New Guinea, the region of East Melanesia (broadly spanning from the island of New Britain to Fiji) is listed as a biodiversity hotspot on the basis of having >0.5% of Earth’s total vascular plants while retaining less than 30% of its original vegetation^8^. However, species richness patterns and correlates are not necessarily congruent among taxa^9^, and the extent to which plant megadiversity in Melanesia is mirrored in other biota remains largely unexamined. Furthermore, patterns of endemism and conservation threat across most of Melanesia are at best coarsely resolved for any taxa.

Amphibians are the most threatened class of terrestrial vertebrates globally^10^, with an estimated 39–41% of species at risk of extinction^11,12^. Recent rapid declines of amphibian populations have been primarily driven by habitat destruction and/or pathogens^10,13^. Juxtaposed against increasing threats, the recognised richness of amphibian species has also increased dramatically—especially in tropical areas^14,15^—with an estimated 25% increase in described species since 2004^16,17^. This inventory work has revealed regions with high concentrations of richness, endemism, and threat, especially in insular frog faunas such as those of Madagascar, Indonesia and Sri Lanka^17^. Unfortunately, in the worst-case scenarios taxa have only been recognised shortly before, or even after, their apparent extinction^18^.

In recent years there has been considerable effort to better document the amphibian fauna of Melanesia, which is comprised entirely of frogs^17,19,20^. Although there has been no synthesis of the diversity and conservation status of Melanesian frogs, the recent recognition of a remarkable concentration of endemic biota, including frogs, in a small portion of that region^21^ suggests that Melanesia’s total frog diversity and endemism could be very high. Here we quantify amphibian richness in Melanesia, place this diversity within a global context, and identify concentrations of richness and threats. Our results show that Melanesia—and especially the island of New Guinea—has the most species-rich insular frog fauna in the world, even though large portions of the region remain unsurveyed or poorly documented and many discovered taxa await description. Distributional data suggest that alpha diversity is highest in lower montane areas, and that small range sizes and high species diversity in a radiation of direct-developing taxa is a key factor underpinning megadiversity. Conservation assessment further highlights geographic concentrations of recently documented small-range species of conservation concern, especially in eastern Papua New Guinea and the Solomon Islands. Nonetheless, by world standards this biota is comparatively intact, providing an unparalleled opportunity to understand and conserve a megadiverse insular amphibian fauna.

## Results and discussion

### The richness of Melanesian Frogs

**Fig. 1:**
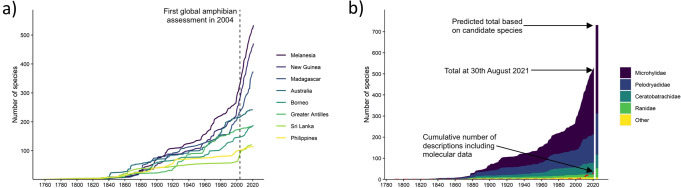
Temporal trends in the documentation of the Melanesian frog fauna. **a** Species accumulation curves for species-rich (>100 species) insular frog biotas (Species lists from AmphibiaWeb as of 1 October 2021). Separate accumulation curves are given for the entire fauna of Melanesia (including New Guinea), and the fauna of New Guinea and nearby predominantly land-bridge islands. **b** Species accumulation curve for frogs within Melanesia. Bar at end indicates predicted number of species in each major family based on known, but as yet undescribed candidate species.

**Fig. 2:**
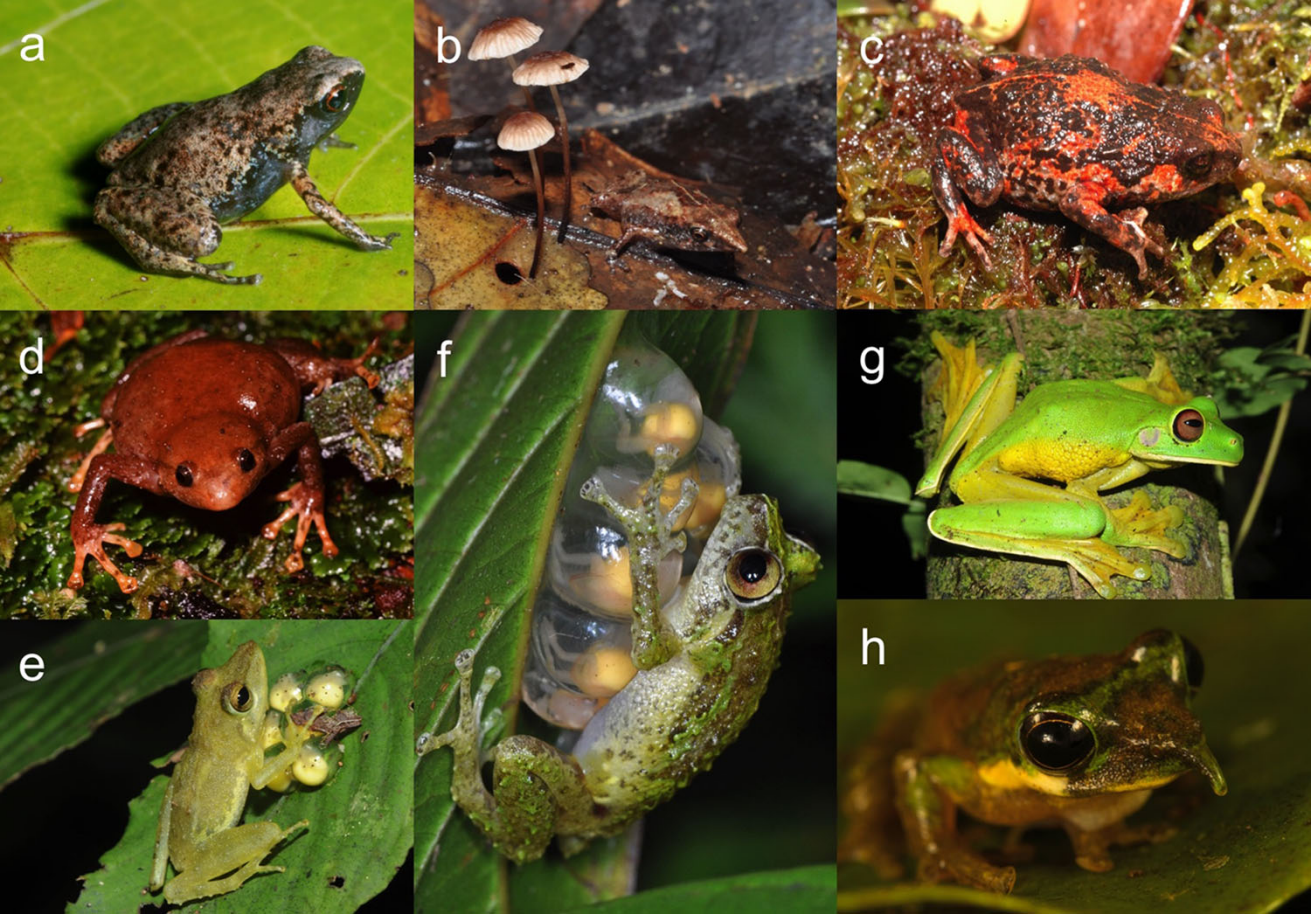
Melanesian frog species described within the last 15 years illustrating the ecological and morphological diversity of the fauna. **a** *Paedophryne titan* and **b** *Choerophryne gracilirostris* – examples of lineages that have undergone convergent minaturisation; **c** *Choerophryne alpestris* – a fossorial species within a largely scansorial lineage; **d** *Xenorhina macrodisca* – scansorial species within a largely fossorial lineage; **e** *Cornufer custos* and **f** *Oreophryne oviprotector* - independent derivations of complex parental care; **g** *Litoria pallidofemora* – extensive digital webbing for parachuting; and **h** *Litoria pinocchio* – sexually dimorphic and erectile rostral spikes. Photographs F. Kraus (**a**), S. Richards (**b**–**g**), and courtesy of T. Laman (**h**).

Approximately 7.2% (534 out of 7404) of Earth’s recognised frog species occur in Melanesia, a region comprising < 0.7% of the world’s land area. Frog richness in Melanesia, and especially on New Guinea and nearby land-bridge islands (471 species), is higher than in any other tropical insular region (Fig. 1a). New Melanesian frog species have been described at an average rate of nearly 13 species/year since 2000, and the recognised frog fauna has grown by > 50% in that timeframe (Fig. 1b). The authorship of new species has been concentrated, with six authors featuring on 20 or more descriptions since 2000, and one or more of these six authors on every species description since 2000. A small number of species descriptions has included genetic data (31 species), although a higher number of Melanesian frog species have at least one sequence available on GenBank (~38%, or approximately 200 species). This taxonomic work has revealed or emphasised many evolutionary novelties (Fig. 2): multiple apparently independent derivations of extremely miniaturised vertebrates^22–24^, including some of the world’s smallest known tetrapods^23^^,^^25,26^; multiple derivations of complex parental care in different genera^27,28^; frequent evolutionary shifts between terrestrial, arboreal and scansorial lifecycles^22,29^; the most extreme sexual size dimorphism yet documented in anurans^30^; drastic ontogenetic colour change^31^; a radiation of canopy-dwelling treefrogs^32^ that show extensive finger webbing and parachuting behaviour convergent with unrelated frog lineages in Asia and the Neotropics; and treefrogs with erectile noses^33,34^. Taxonomic work has also elucidated novel concentrations of range-restricted endemic taxa, especially in the Milne Bay Region at the far eastern edge of New Guinea^21^.

Frog species richness in Melanesia is highly concentrated into just three families, with Pelodryadidae (137 recognised species, estimated ~200) and especially Microhylidae (317 species, estimated >400) dominating. Melanesian Pelodryadidae are phylogenetically interdigitated with relatives in Australia, suggesting multiple dispersals between the two regions^35^. In contrast, ancestors of the direct-developing microhylids colonised Melanesia from Asia via trans-marine dispersal likely only once^36^, radiated across open ecological niches^37^, and are now the most species-rich insular radiation of frogs in the world. The third major family comprises an ecologically diverse radiation of the direct-developing Ceratobatrachidae (57 species, estimated 66) largely associated with island-arc terranes of East Melanesia and the Philippines, indicating a long history of insular diversification and trans-marine dispersal^38^. The predominance of direct-developing frogs in Melanesia (~70% of species) mirrors insular faunas in Madagascar (~34%), Sri Lanka (~67%) and the Greater Antilles (~87%). The other four frog families in Melanesia are all relatively species poor (2, 3, 4, and 13 species) (Fig. 1a), centred in New Guinea, and include lineages originating in Asia (Ranidae, Dicroglossidae) or Australia (Myobatrachidae, Limnodynastidae).

The described diversity of Melanesian amphibian species remains an underestimate. Survey work and investigation of museum collections by the co-authors identified ~190 additional candidate species distributed across 16 different genera, mostly from Papua New Guinea, suggesting a total richness of over 700 frog species (Fig. 1a, Supplementary Table 1). This estimated percentage of undescribed diversity (~25%) mirrors estimates for the New Guinean flora (~18–22%)^7^. The majority of candidate species are concentrated in the two most diverse families (Microhylidae and Pelodryadidae), although genetic, morphological, and acoustic evidence indicate the diversity of Melanesian Ranidae is also underestimated (S. Richards and F. Kraus pers. obs.). Most material documenting candidate species has been collected in the last 20 years, and the vast majority is from Papua New Guinea (Supplementary Fig. 1). There is some suggestion of a slowing in the rate of candidate species discovery in the last decade (Supplementary Fig. 2); however, several of the most active field workers in this region have ceased survey work in recent years, which likely accounts for much of this decline. The pervasiveness of complexes of morphologically and/or acoustically cryptic taxa is poorly understood; survey work continues to reveal novelties, and large areas of the region remain unsurveyed or undersampled. In particular, comparisons of area-to-diversity ratios between the better-known eastern portion of New Guinea (Papua New Guinea) with the poorly surveyed western (Indonesian) portion of the island further suggest that, even with candidate species included, diversity in the latter region may be underestimated by as much as 50% (Supplementary Methods and Results, Supplementary Table 2). These trends and patterns all indicate that ~ 700 species is a very conservative minimum estimate of total diversity and support analyses in other taxa showing Melanesia remains a hotspot of unrecognised diversity^39,40^.

### Endemism and distributional patterns

The Melanesian frog fauna is highly endemic (97.2%), with tiny proportions of species shared with Australia (2.4%) or with islands farther west in Indonesia (0.6%), indicating that Australia and Melanesia are discrete centres of frog diversification, despite periodic connection via land bridges through the late Tertiary^41^. The vast majority of Melanesian frog species (471) occur on New Guinea and nearby land-bridge islands (Raja Ampats, Japen and the Milne Bay islands). In comparison, the frog fauna of the much smaller region of Maluku is depauperate (16 species, of which nine are endemic) but also almost certainly underestimated (e.g., there are no Microhylidae recorded from Buru). Most taxa from Maluku are congeneric (and several conspecific) with lineages centred on New Guinea, supporting the biogeographic clustering of Maluku’s amphibians with the main island of New Guinea. In contrast, the frog fauna of East Melanesia is more diverse and highly endemic and dominated by an ecologically diverse radiation of a different family (Ceratobatrachidae) with only four (all pelodryadid treefrogs) out of 56 species shared with nearby New Guinea. East Melanesia and New Guinea appear to be discrete and long-isolated centres of diversification, as expected from their independent geological histories^42^.

**Fig. 3:**
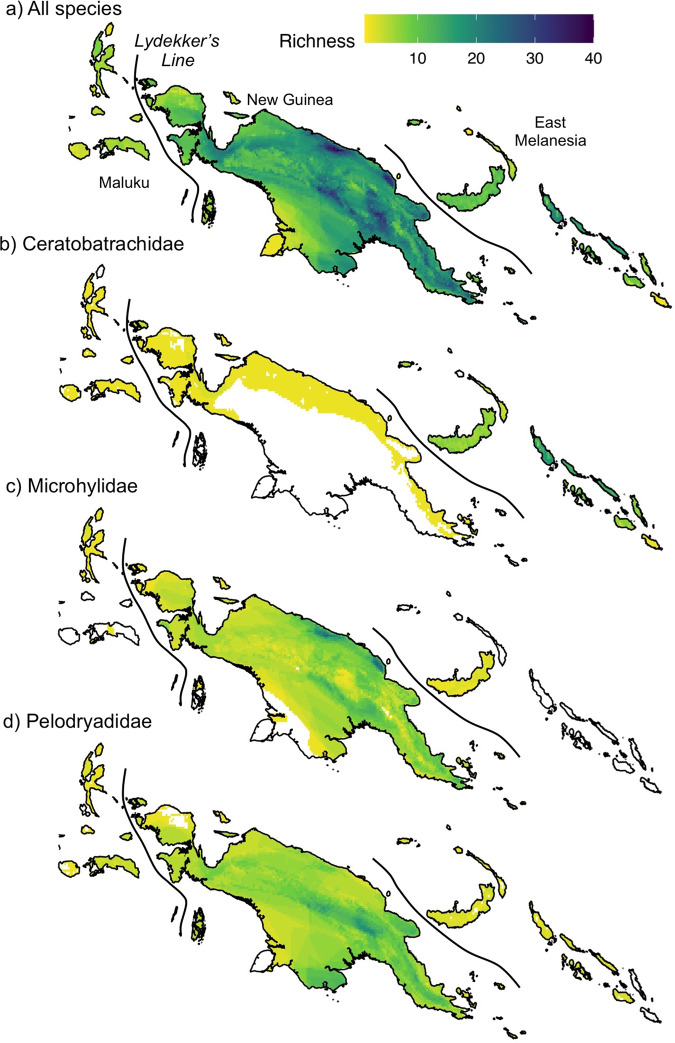
Frog species richness in Melanesia based on IUCN distributional maps for all species described by 2019. **a** All species; **b** Ceratobatrachidae; **c** Microhylidae; **d** Pelodryadidae. Areas of highest estimated diversity correspond to mountain ranges in central and northern New Guinea. The boundaries between Maluku, New Guinea and East Melanesia are indicated. Fiji has only two frog species and is geographically distant from other areas of Melanesia inhabited by frogs and is not visble on this map.

Melanesia spans five countries, and this has possibly to some degree masked the exceptional species diversity of the overall region. Papua New Guinea has the highest number of species (398) and endemic species (318). This likely reflects some combination of its slightly larger area (when islands to the north are included), more diverse geological origins, and greater inventory work than seen in neighbouring regions of Indonesia^7^. Papua, West Papua and Maluku (Indonesia) have many fewer documented species (197), of which a majority (134) is endemic. The boundary between Papua and Papua New Guinea is visible in species-richness maps (Fig. 3a), with lower diversity to the west, indicating that the distribution and diversity of frogs in Indonesia remain less documented in science. The frog faunas of the Solomon Islands (21 species) and Fiji (two species) are more depauperate but include a significant endemic or near-endemic component, whereas the geographically intervening islands of Vanuatu support no native frogs.

Based on distribution maps generated for all species recognised by 31 August 2019, the highest regional alpha diversity of frogs occurs along the Central Cordillera of New Guinea (especially in Papua New Guinea) and around the higher mountain ranges along the north coast of Papua New Guinea (Fig. 3a). These centres of diversity correlate with extensive areas of hill and montane forest and broadly correspond with elevational species-richness patterns for mammals and birds in Melanesia^43^ and for many other taxa elsewhere in the tropics^44,45^. Large areas of montane forest with lower species richness along the northern versant of the Central Cordillera in Papua New Guinea and in mountain ranges across Papua certainly reflect inadequate sampling. The ceratobatrachid-dominated frog fauna of East Melanesia is richest in Bougainville (Fig. 3d), with attenuating richness towards the west and especially to the east. The two most speciose families both show alpha diversity peaks in mountainous areas of central New Guinea (Fig. 3c–d). In contrast, microhylids are largely absent from the seasonally dry woodlands of the Trans-Fly region in southern New Guinea and exhibit high diversity in northern New Guinea, whereas pelodryadids are much more speciose in the lowlands of southern New Guinea than northern New Guinea. These broad trends may have both ecological (sensitivity of direct-developing microhylids to dry conditions) and historical (Australia as a centre of origin for savanna-adapted Pelodrydidae) underpinnings.

The historical and contemporary factors underpinning high frog species diversity in New Guinea remain largely unstudied, especially when compared to other species-rich insular amphibian faunas such as Madagascar^46^ or the Greater Antilles^47^. When compared to some areas of the Neotropics, alpha and beta diversities of frogs in lowland forests in the basins of the Sepik and Ramu rivers in New Guinea are unremarkable^48^. However, the Milne Bay Region has exceptionally high levels of endemism^21^, so species turnover will be higher in this area. Extent-of-occurrence estimates derived from IUCN maps indicate that direct-developing microhylids have smaller mean and median range sizes than all other families of frogs in Melanesia (Supplementary Table 3). Microhylidae also dominate anuran species diversity in Milne Bay^21^ and many mountain areas where standing water is very limited^49^. These data suggest that, as with some areas in the Neotropics^50^, high beta diversity in lineages with direct development is a key factor underpinning amphibian megadiversity in Melanesia. To address these questions further, synthetic analyses are required to better quantify the extent to which regional megadiversity in Melanesia reflects high community diversity versus species turnover, how elevation and insularity moderates these two parameters, and to what extent emergent patterns may differ from diverse frog communities in other regions such as the Neotropics.

### The conservation status of Melanesian Frogs

**Fig. 4:**
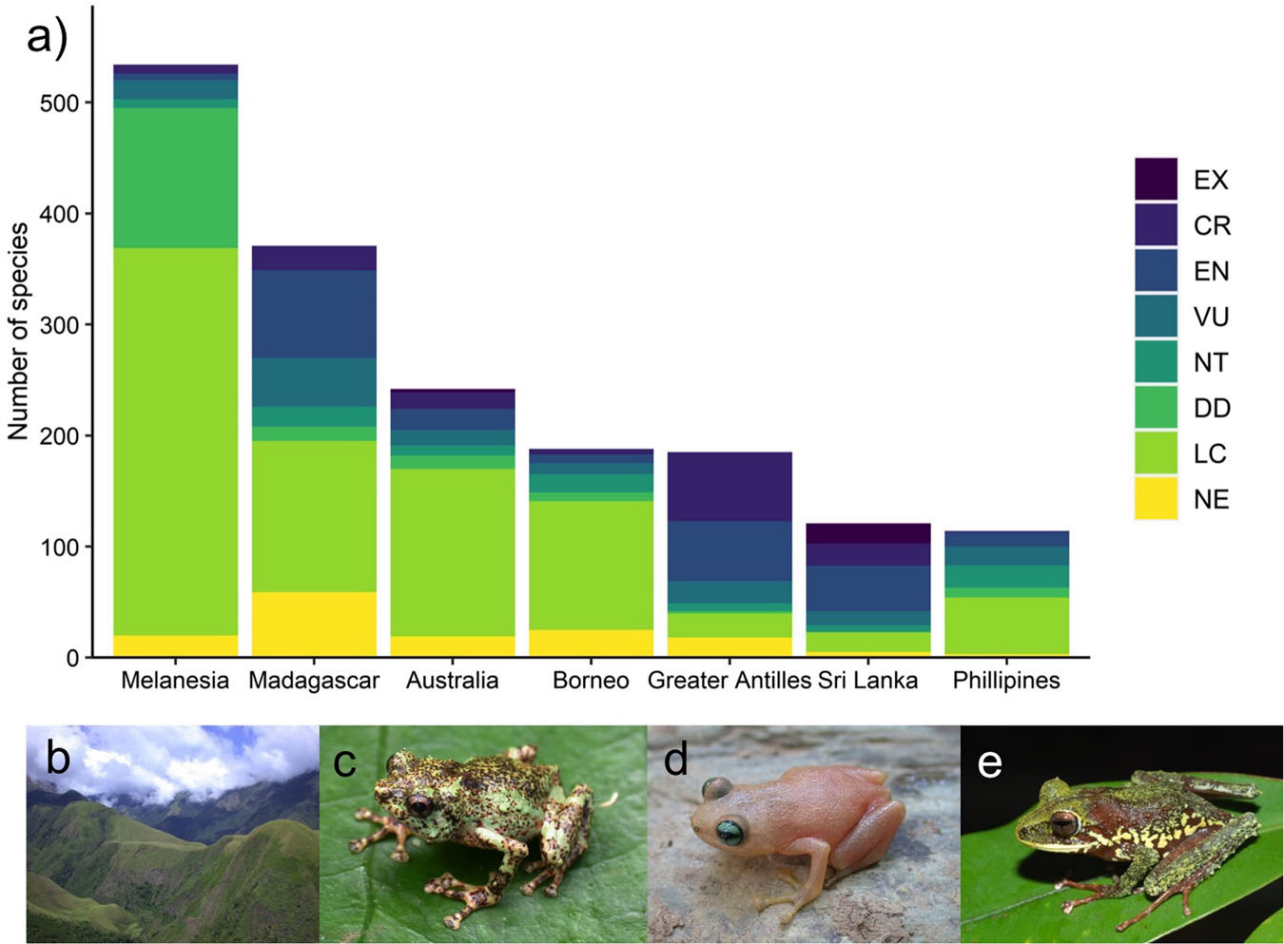
The conservation status of Melanesian frogs. **a** Comparison of number of species in each IUCN threat category across Melanesia, other diverse insular regions, and the nearby continent of Australia. Melanesia has a proportionally low number of threatened taxa but high number of Data Deficient taxa (EX Extinct, CR Critically Endangered, EN Endangered, VU Vulnerable, NT Near Threatened, DD Data Deficient, LC Least Concern, NE Not Evaluated); **b** Slopes around Mt Simpson, Milne Bay Province, a hotspot of threatened frog diversity due to forest loss through conversion to anthropogenic grasslands; **c** *Choerophryne sanguinopicta* from Mt Simpson (Critically Endangered); **d** *Oreophryne ezra* from Rossel Island (Critically Endangered) and; **e** *Cornufer citrinospilus* from New Britain (Vulnerable). Photographs F. Kraus (**b**–**d**), S. Richards (**e**).

The frog fauna of Melanesia is currently less threatened but more Data Deficient than other comparable insular regions (Fig. 4a). The vast majority of Melanesian frogs are categorised as Least Concern (68%) or Data Deficient (24%). Thirty-one species (6%, or 8% if Data Deficient taxa are excluded) are threatened (Critically Endangered, Endangered, Vulnerable) (Supplementary Table 4), and eight species are considered Near Threatened. No species are assessed as Extinct or Extinct in the Wild. Since the first Global Amphibian Assessment in 2004, the number of Melanesian frog species has grown by 44%, and nearly 60% of the 31 Melanesian frog species now considered threatened were described after 2004 (Fig. 1a). Only one change in status between 2004 and 2019 was considered genuine (*Cophixalus sphagnicola*), due to the emerging threat of a newly opened mine. All other status changes (for 116 taxa) reflect better information on distribution or changed assessment protocols (Supplementary Table 5). Applying stricter criteria for use of the Data Deficient category in the 2019 IUCN assessment reduced the number of Data Deficient species when compared to 2004 (125 versus 197), but Melanesia still has a higher percentage of Data Deficient taxa than other species-rich tropical insular faunas (Fig. 4a).

All Critically Endangered and Endangered—and most of the Vulnerable—species were listed because of their small extent of occurrence and on-going decline in habitat area and/or quality (criteria B1ab(iii)) (Supplementary Table 6). The key threatening processes were typically forest disturbance or loss due to conversion to plantations or gardens, repeated burning, or mining (Fig. 4b–c). Only two insular species with very localised montane distributions were considered threatened by climatic disturbance and/or climate change alone (*Cornufer citrinospilus* and *Oreophryne ezra*) (Fig. 4d–e). No species were currently declining from pathogens, and in particular *Batrachochytrium dendrobatidis* (*Bd*), which remains undetected in Melaneisa^51^. However, the introduction and establishment of *Bd* has been identified as a severe threat for well over one hundred taxa^52^, especially for montane pelodryadid treefrogs, a group that has been devastated by this disease in parts of Australia.

**Fig. 5:**
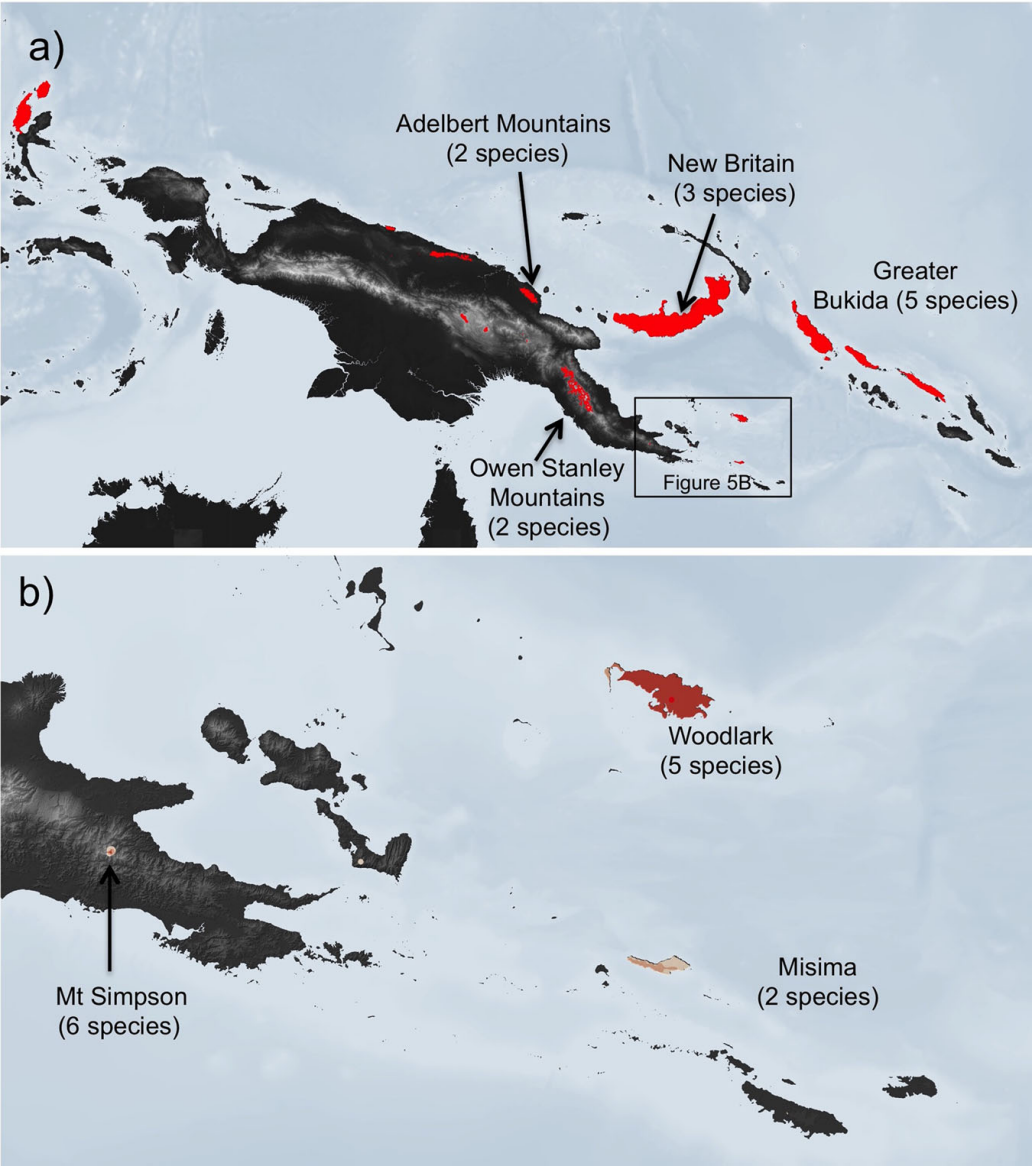
The distribution of threatened frogs in Melanesia. **a** The estimated distribution of all 31 Melanesian frog species considered Critically Endangered, Endangered or Vulnerable at the end of 2019. Distributional areas are not colour coded by the number of threatened taxa. **b** Close up of the Milne Bay endemism hotspot. Distributional areas are colour coded by number of taxa, with darker tones indicating more taxa. In both **a** and **b** upland areas or islands where the distributions of two or more threatened species overlap are labelled and the number of threatened taxa are indicated in parentheses. Background maps uses the Shuttle Radar Topography Mission (SRTM) 30-meter digital elevation model, accessed from USGS Earth Explorer (https://earthexplorer.usgs.gov/).

Although much of New Guinea has historically been considered a ‘wilderness area’ with comparatively little human impact^53^, the distributions of threatened taxa also highlight areas of conservation concern wherein range-restricted (often single-island endemic) taxa overlap with extensive and increasing anthropogenic impacts (Fig. 5a–b). Nearly half the species identified as threatened (13) are restricted to a recently delineated dramatic centre of herpetofaunal endemism in the Milne Bay Region at the eastern tip of Papua New Guinea^21^. Three clusters of small-range endemics in this region (all documented in the last two decades) present immediate conservation issues. The first is Mount Simpson, where six microhylids (four named, two awaiting description) with highly restricted ranges are threatened by habitat loss, especially repeated burning and associated conversion of forest to grassland (Fig. 4b). The second is Woodlark Island, where the status of seven endemic microhylids (six named, one undescribed) is likely to worsen rapidly if current, approved proposals to convert large areas of primary forest to oil-palm plantation and/or gold mines proceed^21^. Finally, Misima Island is home to four endemic microhylids (two considered threatened) with ranges that overlap areas disturbed by mining and forest loss^21^. Other regions with multiple overlapping threatened taxa are the Adelbert Mountains in Morobe Province (two species), New Britain (two lowland species and one highland species), and Greater Bukida in the Solomon Islands (three lowland species). These clusters of narrow-range taxa highlight important—and in most cases largely overlooked—conservation priorities for Melanesian frogs (and likely other taxa as well^21,54^). The high percentage of Data Deficient species and low level of survey effort in many areas (especially Papua and West Papua Provinces, Indonesia) also raise the possibility that other threatened hotspots remain overlooked. One area of particular concern may be the island of Biak in Indonesia, which has lost much of its primary vegetation but is home to at least three endemic frogs (one Data Deficient, two Least Concern).

### Understanding and conserving a megadiverse biota

The Melanesian flora and frog fauna are both now shown to be megadiverse and highly endemic, yet both also remain poorly known with large areas under-surveyed. An updated comprehensive assessment of threats and taxonomic trends across the frog fauna presented here further highlights that the biota of Melanesia remains relatively intact and less threatened when compared to other biodiverse insular regions. However, a large proportion of the fauna remains Data Deficient or undescribed, and key hotspots of endemism have been overlooked and are increasingly threatened. In both plants and anurans much scientific knowledge of Melanesia’s biota has also been contributed by a relatively small number of productive, but later-career researchers based outside of Melanesia^7^.

Further documenting and conserving the exceptional diversity of Melanesia presents a suite of challenges and opportunities. Recommendations to enable improved documentation of plant megadiversity in Melanesia^7^ centre around training, capacity-building and support for taxonomy in Melanesia and globally, improving access to specimen collections and diagnostic resources, and ongoing support for survey and collecting within Melanesia. These recommendations apply equally to amphibians. However, addressing these challenges is tempered by the limited career opportunities available to ecologists and taxonomists (both in developed, but especially in developing countries), the variable quality of scientific infrastructure that exists across the region, and the high cost of doing fieldwork in remote areas with limited logistical infrastructure. In the context of these challenges, we hereby focus on suggesting some short-term key priorities and opportunities to build capacity for understanding and conserving frog biodiversity in Melanesia.

First, over the last twenty years opportunities to employ Melanesian nationals in survey, monitoring and outreach work have been (and will continue to be) generated predominantly by NGOs, universities and large-scale extractive projects, for example through recent work in the gas fields of the Papua New Guinea Highlands^49^. While there are diverse perspectives on extractive industries, monitoring and survey work associated with large development projects are a key source of funds to provide training to enable Melanesians to undertake biodiversity work within the region. A key driver of this is strong environmental legislation required by some governments and major lending agencies, in particular the International Finance Corporation under Performance Standard 6^55^. These requirements need to be maintained, enforced and, where possible, exceeded.

To further support fieldwork by national scientists there is a need for more readily accessible identification resources for Melanesian researchers, land-owners and managers. An up-to-date comprehensive identification guide to the frog fauna of the whole region would assist and promote taxonomic, ecological and conservation research. However, for many Melanesians, small, regionally focused guides are more usable. These have already been produced for several areas (Supplementary References), providing a model that can be updated and transferred to other regions. Mobile phones are widely used throughout Melanesia, so app- and online-based identification resources may become increasingly accessible. Smartphone-friendly citizen science platforms like iNaturalist^56^ or even Facebook groups^57^ also provide potentially powerful resources through which locally collected data can be captured, vetted and disseminated, although their use is currently limited in Melanesia due to patchy internet coverage in many areas. Working with and supporting people from Melanesia to explore and increase the use of these resources could help to ensure longer-term preservation and accessibility of species records and associated data.

The latest IUCN assessment for Melanesian frogs also highlights how taxonomic and conservation knowledge is accumulating rapidly. The key geographic areas of threat identified in our study were largely invisible to assessments made less than two decades ago (in 2004) both because the relevant taxonomic work had not been done, and because the situation in Melanesia is changing rapidly. To keep track of these rapid changes it is critical for workers in the region to work together to synthesise and collate new taxonomic, distributional and conservation data. Indeed, since the 2019 IUCN assessment over 20 additional species of Melanesian frogs have been described, and their conservation status should be assessed as a matter of urgency. Preliminary conservation assessments against IUCN criteria are increasingly being included in descriptions, and this trend should be supported and encouraged. More Melanesian nationals need to be involved in conservation assessment processes. Updated comprehensive conservation assessments of other vertebrate groups will also identify complementarity of conservation priorities among taxa in the Melanesian region.

Patterns of distribution and threat suggest some geographic priority areas for documenting the diversity of amphibians (and potentially other low-vagility taxa) in Melanesia. First, work in eastern New Guinea has allowed the delineation of geographically localised clusters of threatened taxa that have until now gone unnoticed, perhaps in part because of the designation of much of Melanesia as a sparsely populated and comparatively undisturbed ‘wilderness’ area^21^. Most threatened frog taxa in these regions are associated with small islands or isolated ‘sky island’ mountains. The degree to which other taxa show endemism in these areas is poorly known. The biotas of potentially comparable islands in Indonesia such as the Raja Ampat Islands, Geelvink Bay and southern Maluku, also remain poorly known, suggesting additional priority areas for survey, taxonomic investigation and conservation assessment. Second, mid-elevation areas show highest alpha diversity, but large areas of this habitat, especially along the northern slopes of the Central Cordillera, remain poorly surveyed. The frog pathogen *Bd* has devastated montane communities of two Australian frog families that also occur in montane New Guinea (Myobatrachidae and Pelodryadidae)^52^. In the unfortunate event that *Bd* colonised New Guinea a wave of rapid declines and extinctions would likely follow^52^, so a strong baseline of information on montane species diversity, distributions and population status is critical for detecting these impacts.

## Conclusions

Our analyses of frogs, and similar work on other taxa^7^, make it increasingly clear that Melanesia, and especially New Guinea, has an exceptionally rich biota and therefore qualifies as a priority region for conservation attention and investment. Melanesia is politically and culturally complex and has a rapidly growing population with often limited access to basic human requirements for health and education. Addressing these developmental needs is critical for effective conservation and is also a prerequisite for generating a pool of individuals with scientific training in biology, but in many cases these needs also result in unprecedented environmental pressures. Here, we highlight previously overlooked areas in Melanesia where there are clusters of endemic species that are already under threat. These areas should be priorities for supporting community-based conservation initiatives. More broadly, by adding further evidence that Melanesia is a remarkable hotspot of biological diversity, we hope to provide a baseline and marker that enables people from across this region to further engage with their natural heritage and, where desired, seek support for its effective management while also achieving their development goals.

## Methods

### Definition of region, species lists and diversity mapping

Our focal region followed the Papuan region of the 2004 Global Amphibian Assessment and included all of Papua New Guinea, the Indonesian portion of New Guinea (Papua and West Papua Provinces), islands of Maluku lying east of Weber’s Line that share multiple genera with mainland New Guinea (especially Buru, Halmahera, Seram and nearby landmasses), the Solomon Islands, and Fiji. To reflect major faunal differentiation within Melanesia, and for the purposes of comparisons with other insular amphibian biotas, we used three subdivisions within Melanesia (Fig. 3a): Maluku, comprising the islands bounded by Weber’s Line to the west and Lydekker’s Line in the east (including Halmahera, Buru, Seram); New Guinea, comprising New Guinea and nearby islands to the west (Raja Ampats), north (Geelvink Bay), south (Aru Islands), and east (Milne Bay); and East Melanesia, smaller islands extending from Manus Island, through New Britain, New Ireland and the Solomon Islands, east to Fiji.

Our species list for diversity comparisons is largely based on the Amphibian Species of the World Database^20^, accessed 1 October 2021), and we sourced IUCN Red List statuses directly from the IUCN’s Species Information Service (SIS) database. We only included native species in our totals. For the conservation assessment, the species list is slightly older (reflecting the total fauna at the time of the most recent conservation assessment in late 2019) and accordingly has 516 species. For the Pelodryadidae we continue to recognise only *Litoria* and *Nyctimystes* pending a species-intensive revision of this family, even though the former genus is clearly paraphyletic^58^. We excluded three additional taxa listed as occurring in the Papuan region by Frost^20^, but which subsequent research has confirmed are not present (*Litoria dahli and Uperolia mimula*) or are human introductions (*Fejervarya cancrivora*).

Despite being a highly endangered taxon of terrestrial vertebrates, new frog species are being recognised at a rapid rate^20^. To understand trends in documentation of species diversity within Melanesian frogs we collated data on the year of description and whether genetic data were used in documenting each species. We then plotted total numbers of species, yearly description rates and use of genetic data as a source of evidence to validate differentiation of species over time. We compared the resulting knowledge-growth trends against similar data for six other regional amphibian biotas, comprising five large islands or archipelagos that are noted for their diverse, highly endemic and/or highly threatened frog faunas (Borneo, Madagascar, Philippines, Sri Lanka and the Greater Antilles) and one isolated continent (Australia). We also plotted species accumulation trends for the highly diverse New Guinean region within Melanesia. To provide a further measure of knowledge state for the Melanesian anuran fauna we used data on Genbank to generate a summary of the percentage of Melanesian frogs for which molecular sequence data is publically available.

We also sought to establish baseline estimates for the number of known, but as yet undescribed, species, and hence provide minimum bounds for the total species richness of the Melanesian frog fauna. Populations were considered candidate species if they show morphological, acoustic and/or genetic differentiation equivalent to, or greater than, described species. Where possible, we also noted the country of occurrence and date of original discovery for all candidate species, and we plotted these to look for trends in the rate and regions in which candidate species are being discovered. We compiled this list from phylogenetic datasets^21,49^, published reports and environmental impacts assessments (some including DNA barcoding results), theses, and species noted through both museum and field-based analyses, but which have not yet been formally described (Supplementary Table 1, Supplementary References). All candidate species have been examined and, in most cases, collected by authors on this paper. We emphasise that in the absence of formal assessment of species boundaries these are estimates, but they are conservative inasmuch as we ignored populations of whose taxonomic status we were less certain.

All compilations of data showed that the diversity of both recognised species and candidate species in the western (Indonesian) portions of Melanesia was much lower than in the eastern portion of Melanesia, despite the similar areal extent of both regions. To provide a coarse estimate of the scale of these inventory gaps in western Melanesia, we calculated area-to-species diversity ratios for the main portion of Papua New Guinea (excluding the East Melanesian islands, with their very different biota). We then used these ratios to generate estimates of likely total diversity of species in the combined region comprised of Papua and West Papua Provinces. We then further used these numbers to estimate the likely number of endemic species in Indonesian New Guinea assuming 80% endemism (the percentage endemism based on recognised species) and 70% endemism (a lower and more likely percentage given that survey work is likely to reveal that many species currently assumed to be endemic to Papua New Guinea, may in the future be found across the border). Estimates of total species diversity and endemic species diversity were then compared with the known values for these parameters (Supplementary Methods and Results, Supplementary Table 2).

We downloaded maps of all assessed frog species from the IUCN Red List website (https://www.iucnredlist.org/resources/spatial-data-download) to generate species-richness maps for Melanesia. From the downloaded shapefiles we generated species-richness raster layers with a resolution of 150 m x 150m for all species. We decided to work at such a high resolution to ensure we were not missing key patterns in species richness. To compare patterns of diversity across families we then generated maps for the three most diverse families (Ceratobatrachids, Microhylidae and Pelodryadidae) and calculated family-specific richness at the same resolution in R (version 4.1.1). The other families present are species depauperate and typically at most contribute one or two species to frog communities in Melanesia. We estimated mean and median distributions for each family using extent of occurrence estimates (EOO) generated by the IUCN.

### Conservation Status Assessment

The second Global Amphibian Assessment of the IUCN Species Survival Commission’s Amphibian Specialist Group provided an updated assessment of the conservation status of Melanesian frogs as of 2019. The IUCN Red List of Threatened Species uses five criteria that provide different indicators of extinction risk: population size reduction (Criterion A); restricted geographic range and decline/fragmentation/severe fluctuation in range or population size (Criterion B); small population size and decline (Criterion C); very small or restricted populations (Criterion D); and probability of extinction from quantitative population analysis (Criterion E). Red List assessments for each species involve collating available published data on these indicators, which are subsequently evaluated by experts. This evaluation serves three functions: to obtain further, often unpublished, information relevant to these indicators; to compare the resulting data against quantitative thresholds to determine whether a species warrants listing in any of the three ‘threatened’ categories (Vulnerable, Endangered, or Critically Endangered); and to identify further research priorities and conservation measures needed. Species accounts and maps are then reviewed post-workshop by IUCN staff in collaboration with experts to ensure accurate capture of available knowledge^50^.

Workshops were held with experts on the systematics, ecology and conservation of Melanesian frogs at the Port Moresby Nature Park and at Queensland Museum, Brisbane. Workshops respectively focused on the frog faunas of Indonesia (17–20 July 2019) and Papua New Guinea (22–26 July 2019, 1–4 August 2019). In total, 10 experts (all are co-authors) with knowledge of the Melanesian amphibian fauna participated. All assessments were reviewed and accepted by the IUCN and are available on the IUCN Red List website (www.iucnredlist.org). For each species identified as threatened, key threatening processes were summarised, and these can be broadly dichotomised into direct anthropogenic disturbance and loss of habitat (forest disturbance or conversion, mining, agricultural expansion) or predicted loss of climatic niche due to anthropogenic warming. We also visualised hotspots of threatened taxa by counting the number of overlapping distribution maps for threatened species (generated by the IUCN) within any given area. We calculated total threatened species richness in grid cells at a ~150 m resolution. To assess the relative conservation status of the Melanesian frog fauna at a global scale, we compared the percentage of taxa in different threat categories with the nearby Australian biota, and five other species-rich insular frog biotas using status data from the IUCN Red List database (31 August 2021).

Since the first Global Amphibian Assessment in 2004 there have been changes in how the Data Deficient and Least Concern categories are applied (www.iucnredlist.org). In the 2004 assessment, many species that were poorly known but occurred in areas without any known threats were listed as Data Deficient. In the 2019 assessment, experts distinguished between genuinely data-poor species for which threats were unknown and those for which no past, present or future threats could be identified or inferred from related parameters, even if they were only known from one or two localities. The former were retained as Data Deficient, whereas the latter were instead considered Least Concern. This resulted in a large number species being moved from Data Deficient to Least Concern (Supplementary Table 5). Comparisons of changes in the number of Data Deficient species need to take these procedural changes into account.

### Reporting summary

Further information on research design is available in the Nature Research Reporting Summary linked to this article.

## Supplementary information

Supplementary Materials

Reporting Summary

## Data Availability

All distributional data generated or analysed during this study are available from the IUCN redlist (https://www.iucnredlist.org/). Summary data on the generic assignment and year of discovery for candidate species are provided in the supplementary material, while specific details on candidate species are available from the authors upon request.
