# Supplementary material for: Melanesia holds the world’s most diverse and intact insular amphibian fauna
Source: Commun Biol. 2022 Nov 4;5:1182. doi: 10.1038/s42003-022-04105-1 (PMC9636264; doi:10.1038/s42003-022-04105-1)
Supplement: Supplementary file 1 — Supplementary Materials [file 42003_2022_4105_MOESM1_ESM.pdf]

Melanesia holds the world's most diverse and intact insular amphibian fauna

Paul M. Oliver[1,2,*], Deborah Bower[3], Peter J. McDonald[4], Fred Kraus[5], Jennifer Luedtke[6,7,] Kelsey Neam[6,7], Louise Hobin[6], Alienor L.M. Chauvenet[1], Allen Allison[8], Evy Arida[9], Simon Clulow[10], Rainer Günther[11], Elizah Nagombi[12], Burhan Tjaturadi[13], Scott L. Travers[14], Stephen J. Richards[6,15]

[1] Centre for Planetary Health and Food Security, Griffith University, Brisbane, Queensland, 4121, Australia

[2] Centre Biodiversity and Geosciences Program, Queensland Museum, South Brisbane, Queensland, 4101, Australia

[3] Zoology Discipline, School of Environmental and Rural Science, University of New England, Armidale, NSW 2351, Australia

[4] Flora and Fauna Division, Department of Environment, Parks and Water Security, Alice Springs, NT 0870, Australia

[5] Department of Ecology and Evolutionary Biology, University of Michigan, Ann Arbor, MI, 48109, USA

[6] IUCN SSC Amphibian Specialist Group, 3701 Lake Shore Blvd W, P.O. Box 48586, Toronto, Ontario, M8W 1P5, Canada

[7] Re:wild, P.O. Box 129, Austin, Texas 78767, USA.

[8] Bishop Museum, 1525 Bernice Street, Honolulu, HI 96817, USA.

[9] Division of Zoology, Research Center for Biology, Indonesian Institute of Sciences (LIPI), Cibinong, Indonesia

[10] Centre for Conservation Ecology and Genomics, Institute for Applied Ecology, University of Canberra, Bruce, ACT 2617, Australia.

[11] Museum für Naturkunde, Berlin, D-10115, Germany

[12] Wildlife Conservation Society, Goroka, Eastern Highlands Province, Papua New Guinea

[13] Center for Environmental Studies, Sanata Dharma University (CESSDU), Yogyakarta, Indonesia

[14] Department of Biological Sciences, Rutgers University-Newark, Newark, NJ 07102, USA

[15] Herpetology Department, South Australian Museum, Adelaide, S.A. 5000, Australia.

* Corresponding author email: p.oliver@griffith.edu.au

Supplementary Methods and Results

Estimating the 'species diversity deficient'

Summary data on both named species and candidate species richness indicated that Papua New Guinea has far more taxa than the western (Indonesian) part of New Guinea. To produce coarse estimates of the extent to which this disparity may be due to underestimation of species diversity in the Indonesian parts of New Guinea due to lack of survey, we extrapolated patterns and trends from better-known Papua New Guinea. First, we calculated ratios of area against numbers of a) recognised, b) candidate and c) the combined total of recognised and candidate species for Papua New Guinea. These area-based ratios were then used to generate a suite of estimates for the likely total species diversity in the combined region of Papua and West Papua Provinces in Indonesia. We then generated estimates of the potential numbers of overlooked endemic species in Indonesia (i.e. predicted number of endemic species minus recognised endemics) under assumptions of 80% and 70% endemism. These percentages of endemism approximate levels of endemism for the two regions based on known taxa (80-82%, see Table S2), and a more conservative 70% based on the premise that further survey will show that some known species occur across international borders.

For calculating land area in Papua New Guinea we excluded the islands of eastern Melanesia, which have a distinctive and largely unique biota, giving a land area of 405,788km^2. The number of recognised species in this area is 335. The land area to species ratio is 1 species per 1211 km^2. For the land area of Indonesia we included Papua and West Papua, but excluded Maluku with its seemingly depauperate insular biota, giving a total land area of 415170 km^2.

These estimates are admittedly coarse, but in all cases suggest the Indonesian species diversity is likely double the current known number of species, and may include between 68–190 as yet unrecognised species that are not in our list of candidate species (Table S2).

Supplementary Table 1: Summary of the generic allocation of candidate frog species from Melanesia known to the authors of this work.

Genus	Est. # candidate species
Austrochaperina	5
Barygenys	2
Callulops	3
Choerophryne	18
Cophixalus	9
Copiula	9
Cornufer	9
Hylophorbus	15
Litoria	47
Mantophryne	2
Nyctimystes	10
Oreophryne	28
Paedophryne	1
Papurana	29
Sphenophryne	1
Xenorhina	5
**Total**	193

Supplementary Table 2. Estimates of frog species diversity in Indonesian parts of New Guinea based on extrapolation of area to diversity relationships in Papua New Guinea (excluding the islands of East Melanesia). The bold numbers represent current numbers of species, candidate species or candidate + recognised species in each of the two main regions. In all categories the predicted total number of species for Indonesian New Guinea (in green) is much higher than the current number of known species. Further analyses based on varying levels of predicted endemism in Indonesia suggest between 68–190 endemic species remain undescribed and undocumented on the Indonesian side of New Guinea.

			Indonesia Endemism Estimates	
	PNG (excl. East Melanesia)	Indonesia (excl. Maluku)	70% endemism	80% endemism
**Land area estimates (sq km)**	405,788	415,170		
**Recognised species**	**335**	**187**		
Endemic recognised species	**269**	**154**		
% endemism	80%	82%		
Ratio of species to area for PNG	1: 1211 km^2			
Indonesia species diversity estimates on PNG ratios		343	240	274
Estimated additional Indonesian species		156	86	120
**Candidate species**	**145**	**25**		
Endemic candidate species	**141**	**23**		
Ratio of species to area	1: 3192 km^2			
Indonesia species diversity estimates on PNG ratios		130	91	104
Estimated additional Indonesian species		105	68	81
**Total Recognised + Candidate**	**480**	**212**		
Ratio of total species to area	1: 964 km^2			
Indonesia species diversity estimates on PNG ratios		431	301	344
Estimated additional Indonesian species		244	147	190

Supplementary Table 3. Mean, standard deviation and median of estimated distribution size for the four most species-rich families of Melanesian frogs derived from extent-of-occurrence estimates generated by the IUCN. The megadiverse microhylids have particularly small ranges, but many taxa remain known only from their type localities.

Name	N	Mean (km2)	SD (km2)	Median (km2)
Ceratobatrachidae	58	17,402	48,640	7,047
Pelodryadidae	132	121,840	443,390	2832
Microhylidae	303	13,216	53,923	426.8
Ranidae	14	178,184	205,259	115,626

Supplementary Table 4. Summary list of Critically Endangered, Endangered, or Vulnerable frogs from Melanesia.

Species	GAA Category	GAA2 category	Region	Range	**Country**
Oreophryne siegfriedi	CR	CR	Sky Islands	Mt Elimbari	Papua New Guinea
Oreophryne sanguinopicta	DD	CR	Sky Islands	Mt Simpson	Papua New Guinea
Cophixalus timidus	DD	CR	Sky Islands	Mt Simpson	Papua New Guinea
Cophixalus misimae	DD	CR	Island	Misima Island	Papua New Guinea
Oreophryne ezra	NE	CR	Island/sky island	Sudest Island	Papua New Guinea
Paedophryne kathismaphlox	NE	CR	Sky Islands	Mt Simpson	Papua New Guinea
Oreophryne penelopeia	NE	CR	Island/sky island	Normanby Island	Papua New Guinea
Oreophryne matawan	NE	CR	Sky Islands	Mt Simpson	Papua New Guinea
Cornufer parkeri	VU	EN	Island	Bougainville	Papua New Guinea
Cophixalus sphagnicola	LC	EN	Sky Islands	Owen Stanley mountains	Papua New Guinea
Choerophryne gudrunae	DD	EN	Sky Islands	Adelbert Ranges	Papua New Guinea
Cornufer parilis	NE	EN	Island	Santa Isabel	Papua New Guinea
Oreophryne cameroni	NE	EN	Sky Islands	Adelbert Ranges, and Torricelli Mountains	Papua New Guinea
Oreophryne picticrus	NE	EN	Island	Misima Island	Papua New Guinea
Litoria rueppelli	VU	VU	Island	Maluku	Indonesia
Sphenophryne rhododactyla	DD	VU	Sky Islands	Owen Stanley mountains	Papua New Guinea
Cornufer wolfi	LC	VU	Island	Bougainville	Papua New Guinea
Cornufer cheesmanae	DD	VU	Mainland	Northern New Guinea	Indonesia; Papua New Guinea
Cophixalus nubicola	VU	VU	Sky Islands	Mt Michael	Papua New Guinea
Cornufer minutus	LC	VU	Island	Bougainville	Papua New Guinea
Cornufer nexipus	DD	VU	Island	New Britain	Papua New Guinea (Bismarck Archipelago)
Austrochaperina novaebritanniae	VU	VU	Island	New Britain	Papua New Guinea
Cornufer desticans	NE	VU	Island	Choiseul and	Solomon Islands

				Santa Isabel	
Choerophryne alpestris	NE	VU	Sky Islands	Central Cordillera	Papua New Guinea
Cophixalus amabilis	NE	VU	Island	Woodlark Island	Papua New Guinea
Cophixalus clapporum	NE	VU	Island	Woodlark Island	Papua New Guinea
Cophixalus tenuidactylus	NE	VU	Sky Islands	Owen Stanley Mountains	Papua New Guinea
Barygenys apodasta	NE	VU	Island	Woodlark Island	Papua New Guinea
Cornufer citrinospilus	NE	VU	Island/sky island	Nakanai Mountains	Papua New Guinea
Mantophryne insignis	NE	VU	Island	Woodlark Island	Papua New Guinea
Oreophryne phoebe	NE	VU	Island	Woodlark Island	Papua New Guinea

Supplementary Table 5. Change in the number of species listed under different IUCN categories between the first Global Amphibian Assessment in 2004, and the second Global Amphibian Assessment in 2019.

	2004	2019
CR	1	8
EN	2	6
VU	13	17
NT	1	8
LC	157	349
DD	197	125
NE	142	-

Supplementary Table 6. Criteria under which frog species were listed during IUCN assessments

	2004		2020	
	B1ab(iii)	D2	B1ab(iii)	D2
CR	1		8	
EN	1		6	
VU	5	7	8	9
NT			1	1
LC			28	6
DD			9	8

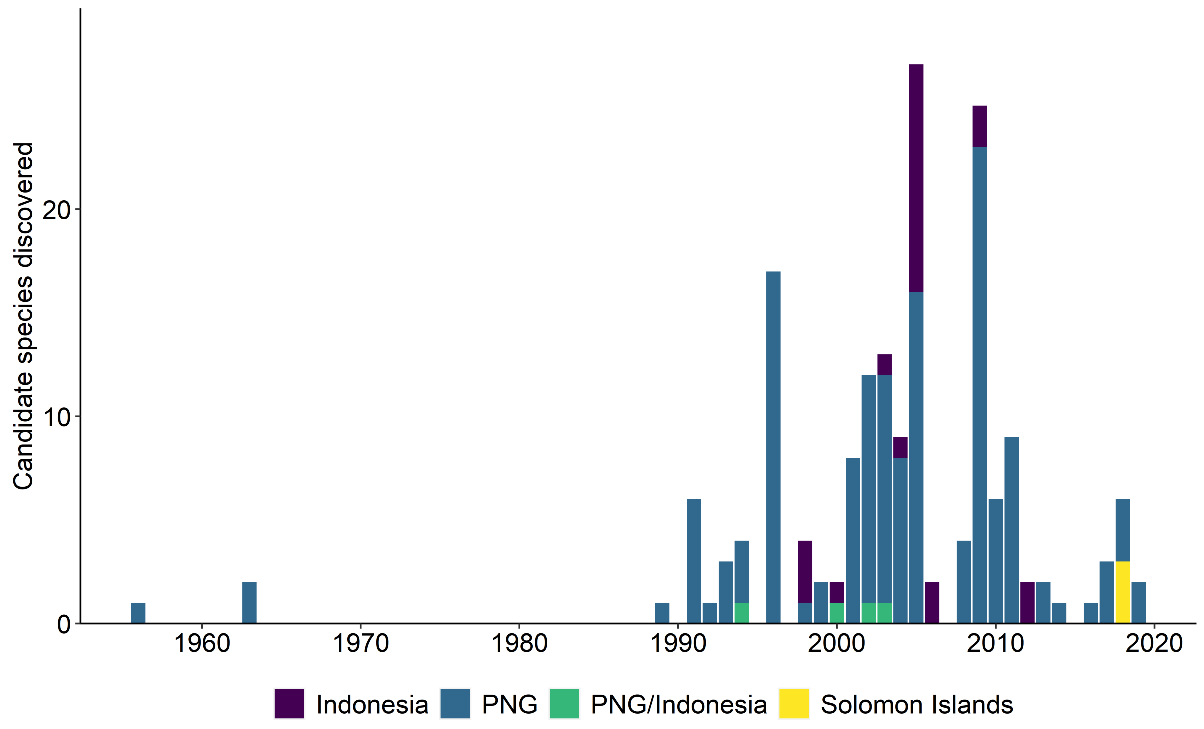

Supplementary Figure 1. Known year of first collection for candidate species of Melanesian frog discovered between 1956-2020, but not yet described at the time this paper was being prepared in late 2021.

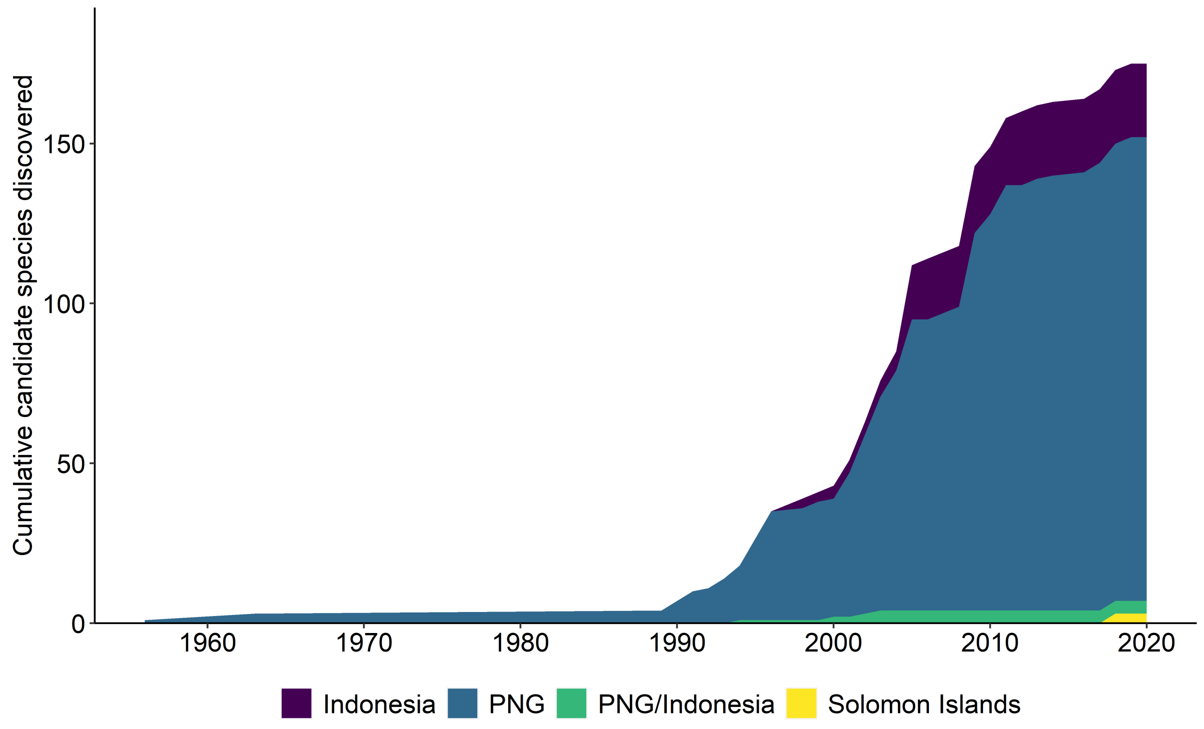

Supplementary Figure 2. Cumulative summary data for candidate species of frog from Melanesia discovered between 1956-2020, but not yet described at the time this paper was being prepared in late 2021.

Supplementary References: Summary list of literature and resources highlighting candidate species of frog from Melanesia. This list does not capture all candidate species in our lists, but highlights the diverse and growing number of studies emphasising that species diversity in Melanesia remains underestimated.

Fieldguides and other books:

Dahl, C. and Richards, S.J. (2021) 'A Pocket Guide to Frogs of Lowland Forests around Kau, Morox, Ohu and Wanang Villages of Madang Province, Papua New Guinea'. (University of South Bohemia Press.)

Richards, S. J. (2018). 'Identification Guide to Flora and Fauna of Hides Ridge and the Agogo Range (Moro), Papua New Guinea'. (ExxonMobil PNG Limited.)

Richards, S. J. (2002). 'Rokrok: An illustrated field guide to frog of the Kikori Integrated Conservation and Development Project area of Papua New Guinea'. (WWF-PNG: Port Moresby.)

Richards, S. J., Tjaturadi, B., Mumpuni, and Puradyatmika, P. (2015). 'Field guide to Frogs of the Mimika region - Papua, Indonesia'. (PT Freeport Indonesia: Mimika.)

Menzies, J. (2006). 'The frogs of New Guinea and the Solomon Islands'. (Pensoft Publishers: Sofia.)

Pikacha, P., Morrison, C., and Richards, S. J. (2008). 'Frogs of the Solomon Islands'. (Quality Print Limited: Fiji.)

Scientific papers

Donnellan, S. C., Aplin, K. P., and Bertozzi, T. (2010). Species boundaries in the Rana arfaki group (Anura: Ranidae) and phylogenetic relationships to other new Guinean Rana. Zootaxa.

Ferreira, F. (2022) Diversité etbiogéographie d'un genre d'amphibien endémique de Nouvelle-Guinée(Anura :Microhylidae:Asterophryninae:Hylophorbus). Masters thesis, Université Toulouse.

Oliver, L. A., Rittmeyer, E. N., Kraus, F., Richards, S. J., and Austin, C. C. (2013). Phylogeny and phylogeography of Mantophryne (Anura: Microhylidae) reveals cryptic diversity in New Guinea. Molecular Phylogenetics and Evolution 67, 600–607. doi:10.1016/j.ympev.2013.02.023

Oliver, P. M., Iannella, A., Richards, S. J., and Lee, M. S. Y. (2017). Mountain colonisation, miniaturisation and ecological evolution in a radiation of direct-developing New Guinea Frogs (Choerophryne, Microhylidae). PeerJ 5, e3077. doi:10.7717/peerj.3077

Travers, S. L. (2019) The evolution and assembly of a Pacific Island frog fauna. PhD Dissertation, University of Kansas.

Rapid Biodiversity Assessment Reports

Allison, A., Bickford, D., Richards, S.J. and Torr, G. (1998). Herpetofauna Pp 58–62 & 156–158 in A. Mack (Ed) A Biological assessment of the Lakekamu Basin, Papua New Guinea. Rapid Assessment Program Working Paper Number 9. Washington, Conservation International.

Richards, S.J., Iskandar, D.T. and Allison, A. (2000). Amphibians and reptiles of the Wapoga River area, Irian Jaya, Indonesia. Pp. 54–57 & 113–120 In: A. Mack and L. Alonso (Eds) A biological assessment of the Wapoga River area of northwestern Irian Jaya, Indonesia. RAP Bulletin of Biological Assessment 14. Washington, DC. Conservation International.

Richards, S.J., Iskandar, D.T., Tjaturadi, B. and Krishar, A. (2002). Amphibians and reptiles of the Yongsu area, Papua, Indonesia. Pp. 73–75 & 162–163 In: Richards, S.J. and Suryadi, S. (Eds) A biodiversity assessment of Yongsu - Cyclops Mountains and the southern Mamberamo basin, Papua, Indonesia. RAP Bulletin of Biological Assessment 25. Washington, DC. Conservation International.

Richards, S.J., Iskandar, D.T. and Tjaturadi, B. (2002). Amphibians and reptiles of the Dabra area, Mamberamo River basin, Papua, Indonesia. Pp. 76–79 & 164–167 In: Richards, S.J. and Suryadi, S. (Eds) A biodiversity assessment of Yongsu - Cyclops Mountains and the southern Mamberamo basin, Papua, Indonesia. RAP Bulletin of Biological Assessment 25. Washington, DC. Conservation International.

Richards, S.J., (2007). Herpetofauna of the Kaijende Highlands, Enga Province, Papua New Guinea. Pp. 40–46 In: Richards, S.J. (Ed) A rapid biodiversity assessment of the Kaijende Highlands, Enga Province, Papua New Guinea. RAP Bulletin of Biological Assessment 45. Washington, D.C. Conservation International.

Richards, S. J. (2011). Herpetofauna of the Nakanai Mountains, East New Britain Province, Papua New Guinea. Pp. 75–80 In: Richards, S. J. and Gamui, B. (Eds.) Rapid biological assessments of the Nakanai Mountains and the upper Strickland basin: surveying the biodiversity of Papua New Guinea's sublime karst environments. RAP Bulletin of Biological Assessment 60. Arlington, VA. Conservation International.

Richards, S. J. and Dahl, C. (2011). Herpetofauna of the Strickland Basin and Muller Range, Papua New Guinea. Pp. 190–197 In: Richards, S. J. and Gamui, B. (Eds) Rapid biological assessments of the Nakanai Mountains and the upper Strickland basin: surveying the biodiversity of Papua New Guinea's sublime karst environments. RAP Bulletin of Biological Assessment 60. Arlington, VA. Conservation International.

Richards, S.J. (2014). Herpetofauna of the Baiyer River Sanctuary area, Western Highlands Province, Papua New Guinea. Pp 57–65 In: Richards, S.J. (Ed.) A Rapid Biodiversity Assessment of the Baiyer River Region, Western Highlands Province, Papua New Guinea. A report to the Mul Baiyer Lumusa District Administration and the PNG Department of Environment and Conservation.

Richards, S.J. (2015). Herpetofauna. Pp 94–102 In: Richards, S.J. and Whitmore, N. (Eds) A rapid biodiversity assessment of Papua New Guinea's Hindenburg Wall region. Goroka, PNG. Wildlife Conservation Society Papua New Guinea Program.

Richards, S. J. and Aplin, K.P. (2015). Herpetofauna of Manus and Mussau Islands. Pp. 31–37 In: Whitmore, N (Ed) A rapid biodiversity survey of Papua New Guinea's Manus and Mussau Islands. Goroka, PNG. Wildlife Conservation Society Papua New Guinea Program.

Richards, S.J. and Armstrong, K. (2017). Frogs. Pp. 53–90 In: Richards, S.J. (Ed) Biodiversity of the PNG LNG Upstream Project area, Southern Highlands and Hela Provinces, Papua New Guinea. Port Moresby. ExxonMobil PNG Limited.

Richards, S.J., Clulow, S., Bower, D. and Georges, A. (2018). Herpetofauna of the Wau Creek proposed Wildlife Management Area, Gulf Province, Papua New Guinea. Pp 77–90 In: Richards, S.J. (Ed.) Rapid biological assessments of Wau Creek, Uro Creek and Lake Kutubu: documenting biodiversity values to promote forest conservation in the Kikori River basin, Papua New Guinea. Port Moresby. ExxonMobil PNG Limited.

Richards, S.J., (2018). Herpetofauna of the Uro Creek Catchment, Gulf Province, Papua New Guinea. Pp 199–209 In: Richards, S.J. (Ed.) Rapid biological assessments of Wau Creek, Uro Creek and Lake Kutubu: documenting biodiversity values to promote forest conservation in the Kikori River basin, Papua New Guinea. Port Moresby. ExxonMobil PNG Limited.

Richards, S.J. and Dahl, C. (2018). Herpetofauna of the Lake Kutubu Wildlife Management Area, Southern Highlands Province, Papua New Guinea. Pp 307–317 In: Richards, S.J. (Ed.) Rapid biological assessments of Wau Creek, Uro Creek and Lake Kutubu: documenting biodiversity values to promote forest conservation in the Kikori River basin, Papua New Guinea. Port Moresby. ExxonMobil PNG Limited.

Richards. S.J, Armstrong, K.N., Nagombi, E. and Dahl, G. (2021). Frogs. Pp 19–51 In: Richards, S.J. (Ed.) Results of the third PMA3 Biodiversity Survey of the PNG LNG Upstream Project Area, 8 August-2 September 2019. Port Moresby, Exxon Mobile PNG Ltd.
